# Supplementary material for: Computational design of constitutively active mutants of Dopamine D2 receptor inspired by ligand-independent activation mechanisms
Source: BMC Biol. 2026 Feb 5;24:53. doi: 10.1186/s12915-026-02542-6 (PMC12930580; doi:10.1186/s12915-026-02542-6)
Supplement: Supplementary file 1 — Additional file 1: Figures S1-S20 and Tables S1-S4. Figure S1. Free energy landscapes of eleven D2R mutants predicted by in silicon residue scanning projected along microswitches. Figure S2. mScarlet-D2R-SmBiT receptor activity upon vehicle or quinpirole stimulation and Receptor expression normalized baseline activity of three different D2R mutants. Figure S3: Mutant expression and ligand response. Figures S4-S19. Strings averaged over hundreds of iterations for all studied D2R systems initiated from the active structure. Figure S20. Mutation-specific allosteric signal pathways from the extracellular to intracellular domains in the mutant T692.39E. Table S1. Total simulation time for each D2R system. Table S2. Local functional microswitches used to characterize the free energy landscapes. Table S3. Experimental structures of active and inactive D2R and D3R used for structural comparison. Table S4. Twelve contact pairs extracted from structural analysis important for the stability of the inactive state with selected residues in bold for in-silico residue scanning. [file 12915_2026_2542_MOESM1_ESM.docx]

Supplementary Information for

**Computational design of constitutively active mutants of Dopamine D2 receptor inspired by ligand-independent activation mechanisms**

Yue Chen *et al.*

*Corresponding author. Email: [lucied@kth.se](mailto:lucied@kth.se)

**This file includes:**

Figs. S1 to S20

Tables S1 to S4


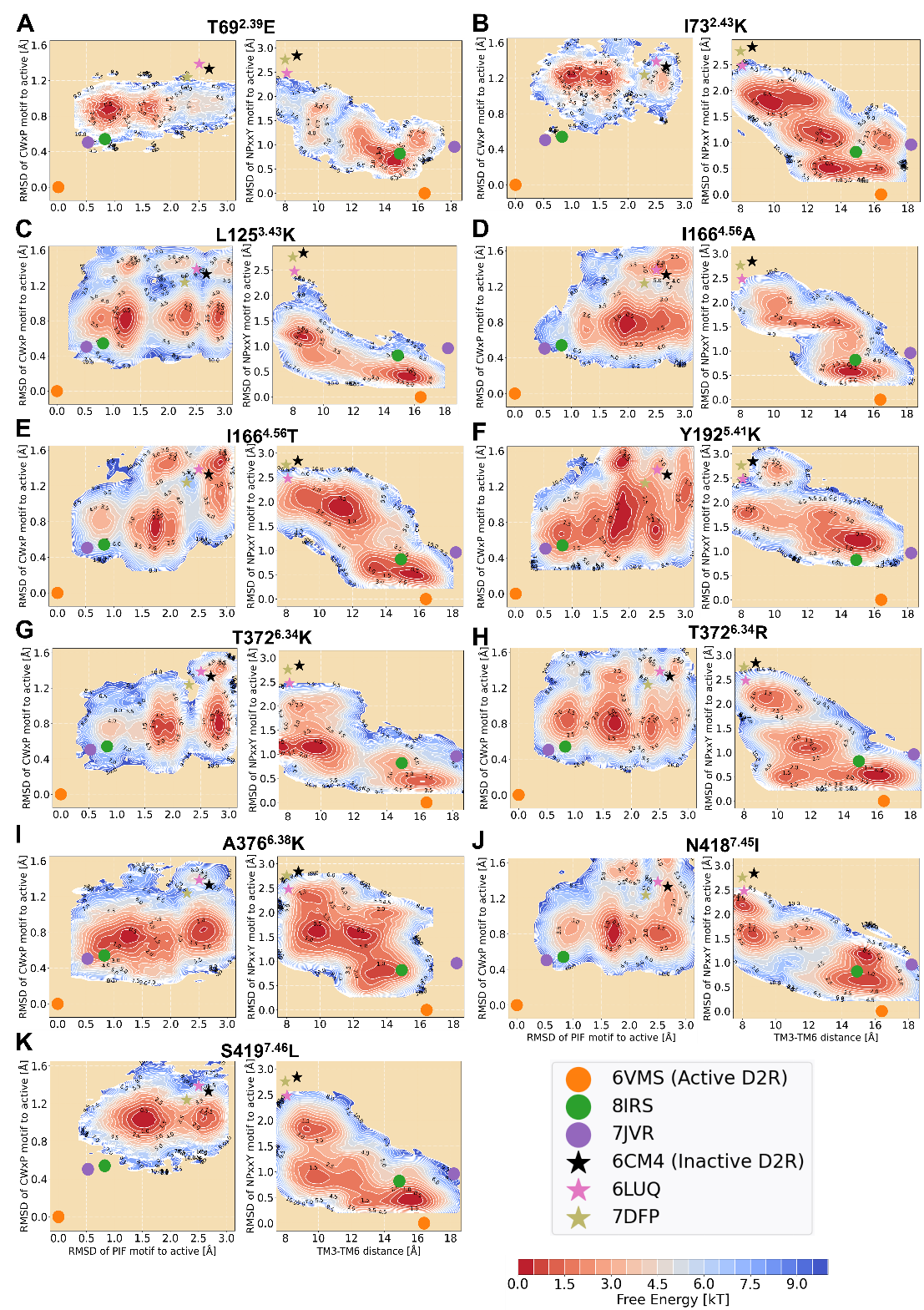


Fig. S1. Free energy landscapes of eleven D2R mutants predicted by *in silicon* residue scanning projected along the RMSD of CWxP motif (measured using heavy atoms RMSD of C385^6.47^, W386^6.48^, and P388^6.50^), the RMSD of PIF motif (connector region: measured using heavy atoms RMSD of I122^3.40^ and F382^6.44^), the RMSD of NPxxY motif (measured using heavy atoms RMSD of N422^7.49^, P423^7.50^ and Y426^7.53^), and TM6 outward movement (represented by the Cα distance between R132^3.50^ and E368^6.30^). Crystal active (PDB codes: 6VMS, 8IRS, and 7JVP) and inactive (PDB codes: 6CM4, 6LUQ, and 7DFP) structures of D2R are depicted by dots and stars, respectively.


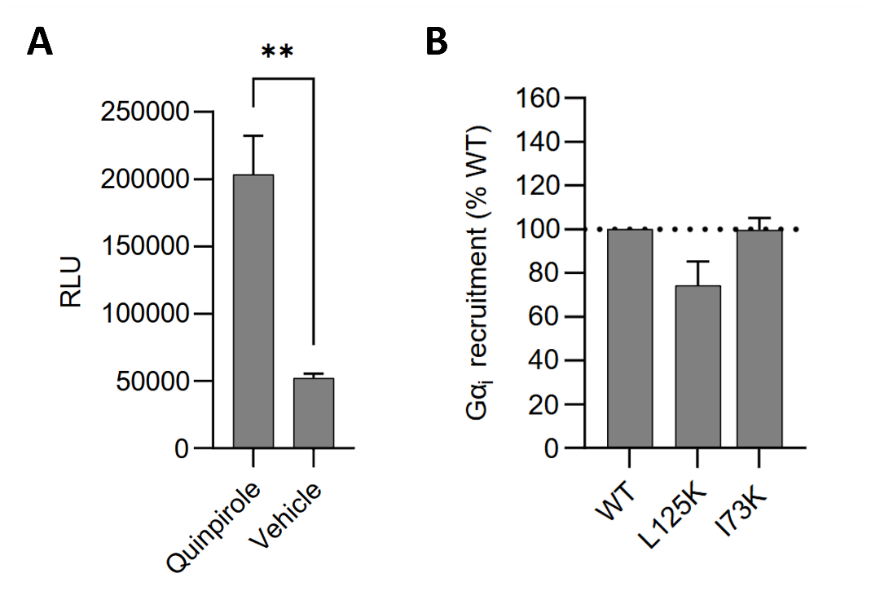


Fig. S2. (A) mScarlet-D2R-SmBiT receptor activity, as measured by the luminescence generated by mini-Gi recruitment, upon vehicle or quinpirole (20 µM) stimulation (n=5 technical replicates) (B) Receptor expression normalized baseline activity of three different D2R mutants (n=6).


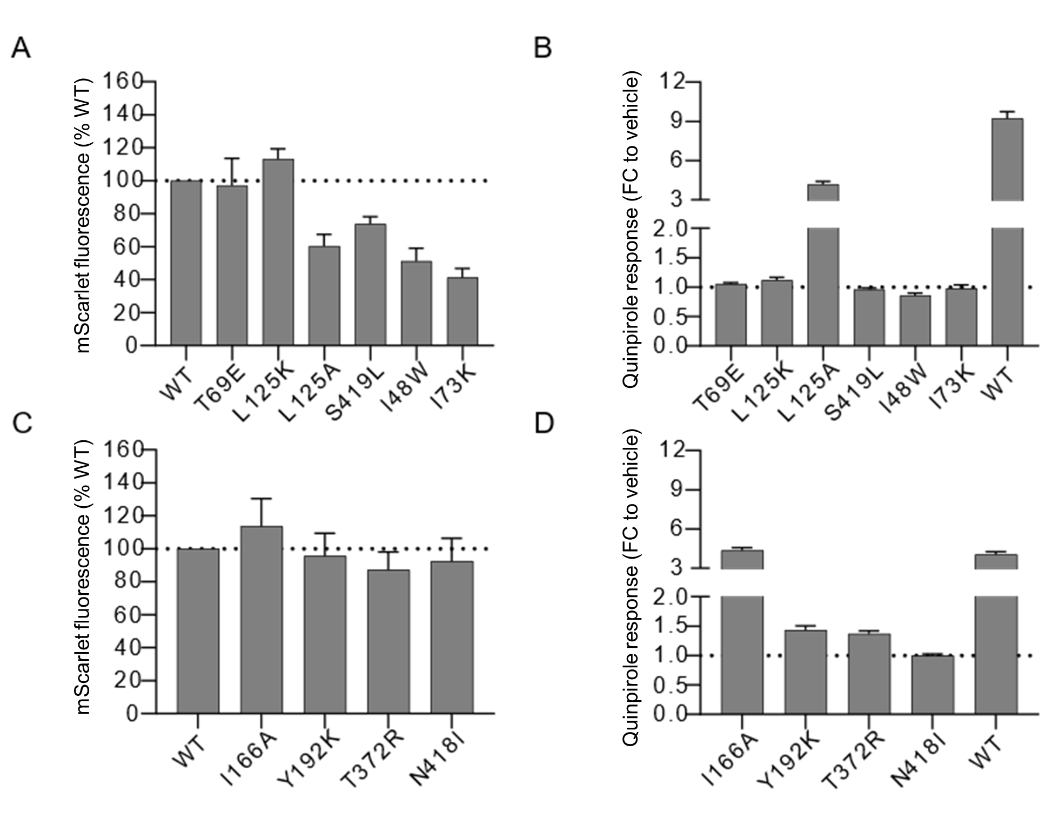


Fig. S3. Mutant expression and ligand response. (A, C) mScarlet fluorescence of WT or single-position mutant mScarlet-D2R-SmBiT constructs expressed in HEK293T cells, normalized to WT (n=4-6). (B, D) Quinpirole response, presented as fold-change relative to vehicle-treated cells, in cells expressing different receptor constructs (n=4-6).


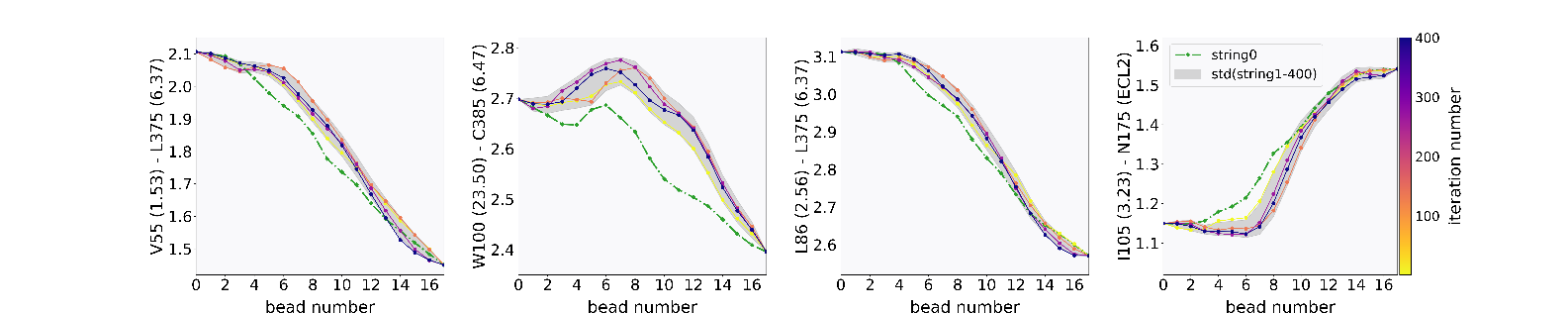


Fig. S4. Strings averaged over hundreds of iterations for unliganded D2R^WT^ initiated from the active structure (PDB ID 6VMS). The top 4 important collective variables (CVs) in Table S1 are shown on the y-axis to evaluate the string convergence. The x-axis shows the evolution of the string points towards the inactive state.


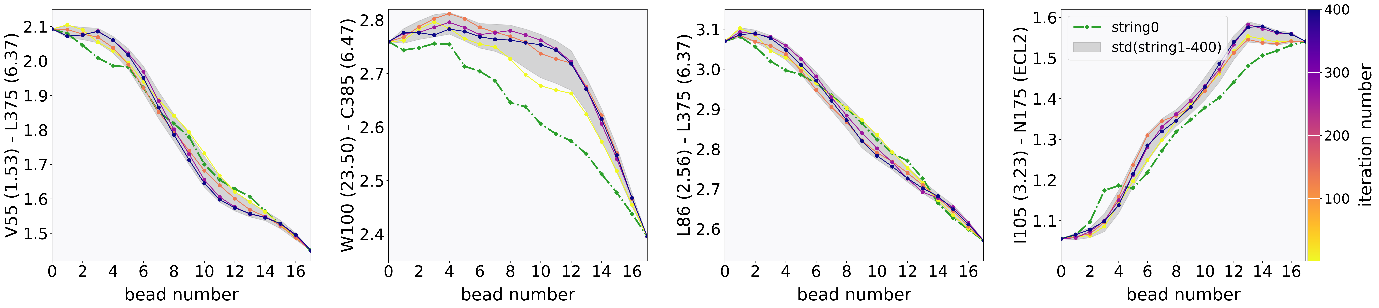


Fig. S5. Strings averaged over hundreds of iterations for unliganded D2R^EM^ initiated from the active structure (PDB ID 6VMS). The top 4 important collective variables (CVs) in Table S1 are shown on the y-axis to evaluate the string convergence. The x-axis shows the evolution of the string points towards the inactive state.


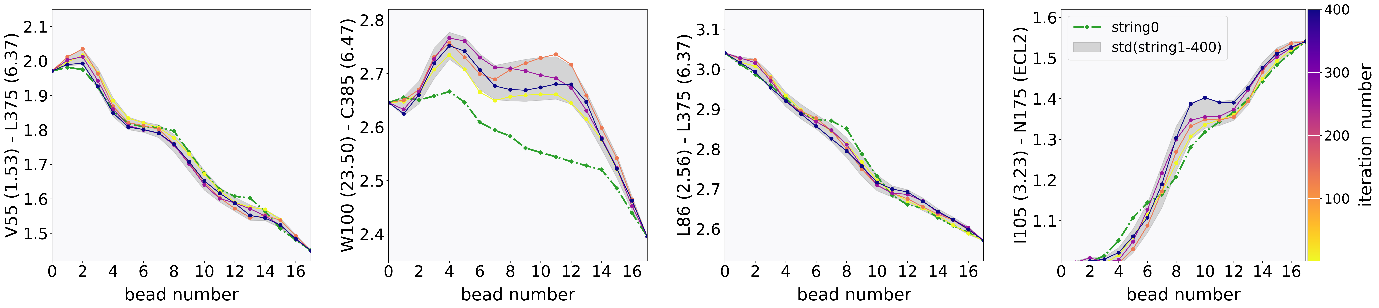


Fig. S6. Strings averaged over hundreds of iterations for unliganded D2R-I48^1.46^A mutant initiated from the active structure (PDB ID 6VMS). The top 4 important collective variables (CVs) in Table S1 are shown on the y-axis to evaluate the string convergence. The x-axis shows the evolution of the string points towards the inactive state.


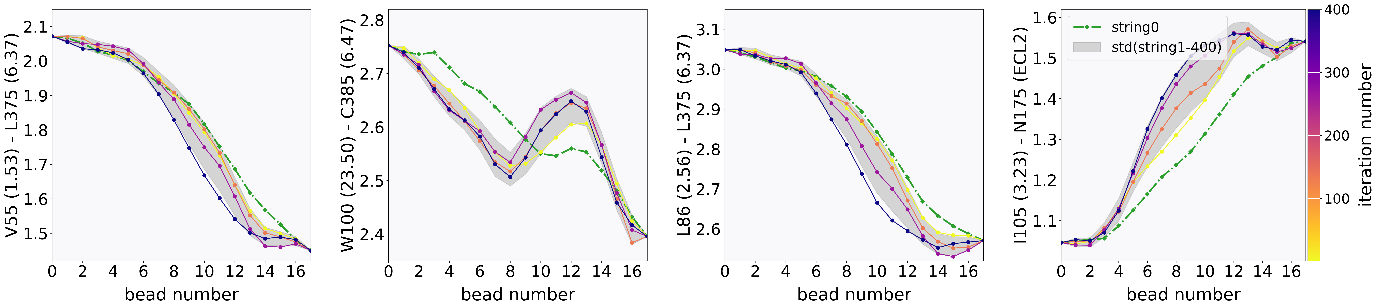


Fig. S7. Strings averaged over hundreds of iterations for unliganded D2R-I48^1.46^Y mutant initiated from the active structure (PDB ID 6VMS). The top 4 important collective variables (CVs) in Table S1 are shown on the y-axis to evaluate the string convergence. The x-axis shows the evolution of the string points towards the inactive state.


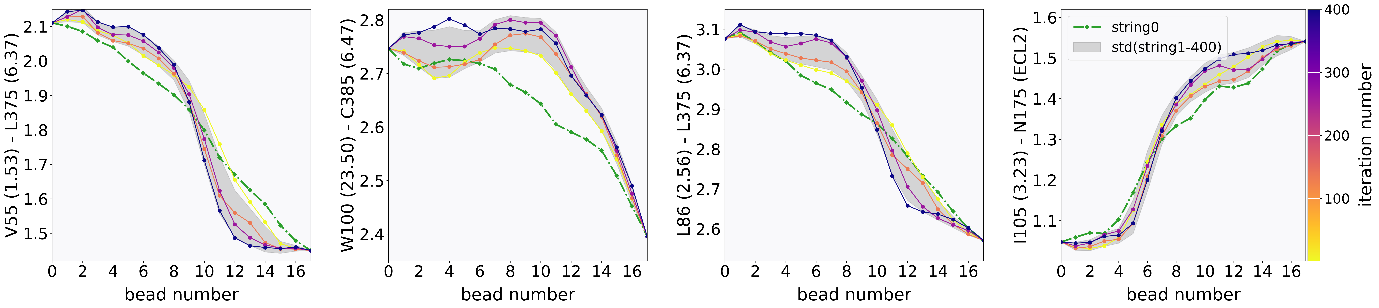


Fig. S8. Strings averaged over hundreds of iterations for unliganded D2R-I48^1.46^W mutant initiated from the active structure (PDB ID 6VMS). The top 4 important collective variables (CVs) in Table S1 are shown on the y-axis to evaluate the string convergence. The x-axis shows the evolution of the string points towards the inactive state.


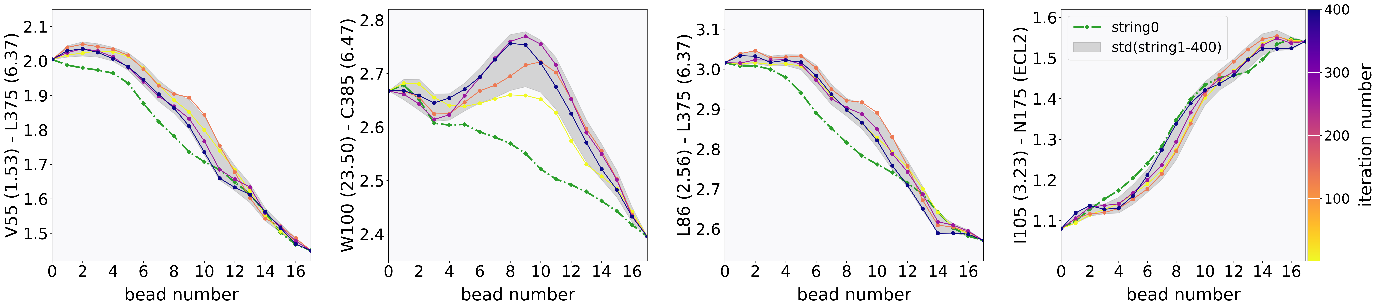


Fig. S9. Strings averaged over hundreds of iterations for unliganded D2R-T69E mutant initiated from the active structure (PDB ID 6VMS). The top 4 important collective variables (CVs) in Table S1 are shown on the y-axis to evaluate the string convergence. The x-axis shows the evolution of the string points towards the inactive state.


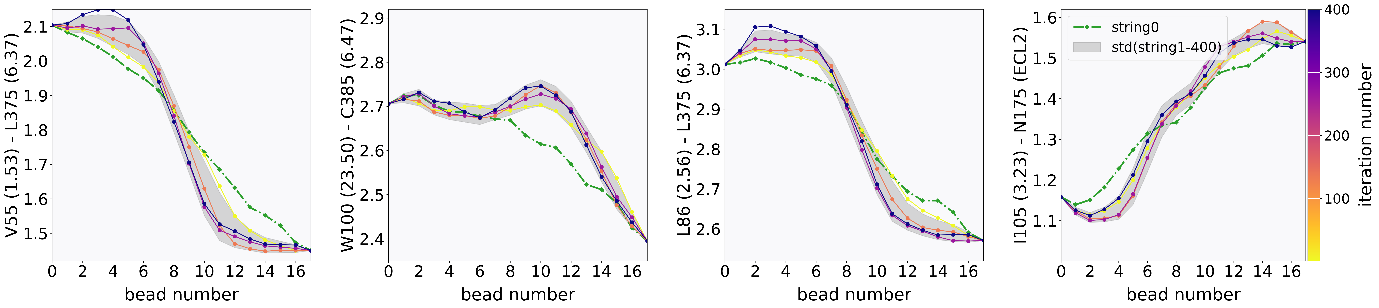


Fig. S10. Strings averaged over hundreds of iterations for unliganded D2R-I73K mutant initiated from the active structure (PDB ID 6VMS). The top 4 important collective variables (CVs) in Table S1 are shown on the y-axis to evaluate the string convergence. The x-axis shows the evolution of the string points towards the inactive state.


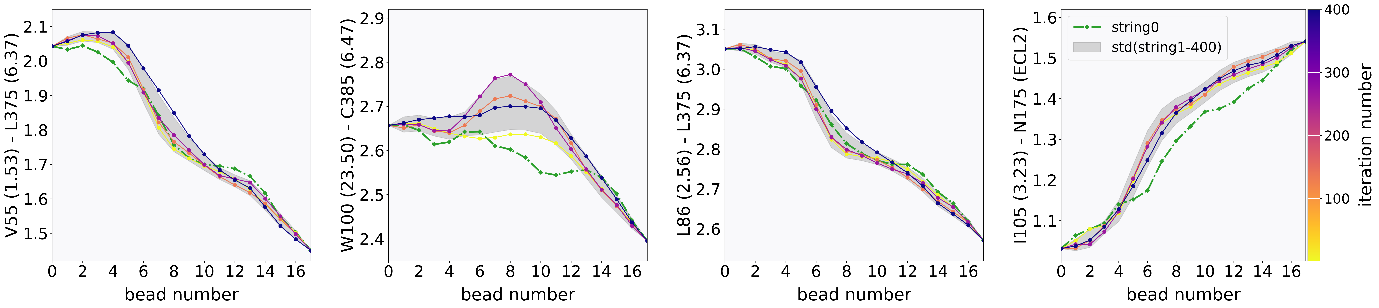


Fig. S11. Strings averaged over hundreds of iterations for unliganded D2R-L125K mutant initiated from the active structure (PDB ID 6VMS). The top 4 important collective variables (CVs) in Table S1 are shown on the y-axis to evaluate the string convergence. The x-axis shows the evolution of the string points towards the inactive state.


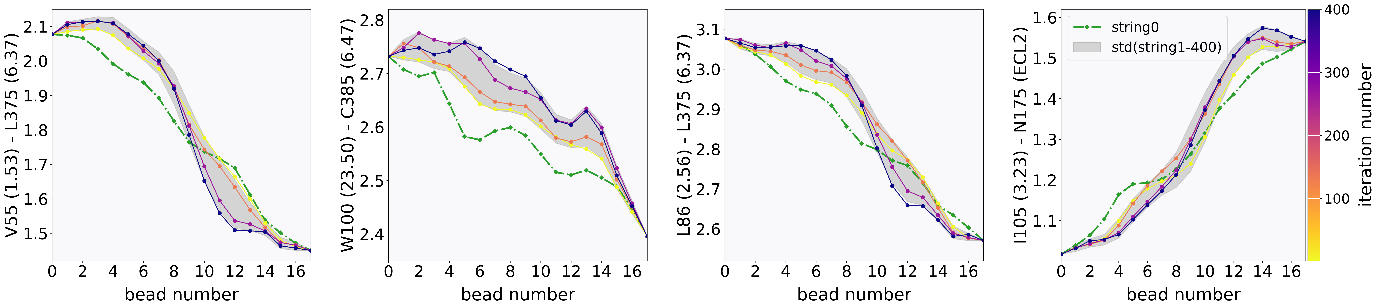


Fig. S12. Strings averaged over hundreds of iterations for unliganded D2R-I166A mutant initiated from the active structure (PDB ID 6VMS). The top 4 important collective variables (CVs) in Table S1 are shown on the y-axis to evaluate the string convergence. The x-axis shows the evolution of the string points towards the inactive state.


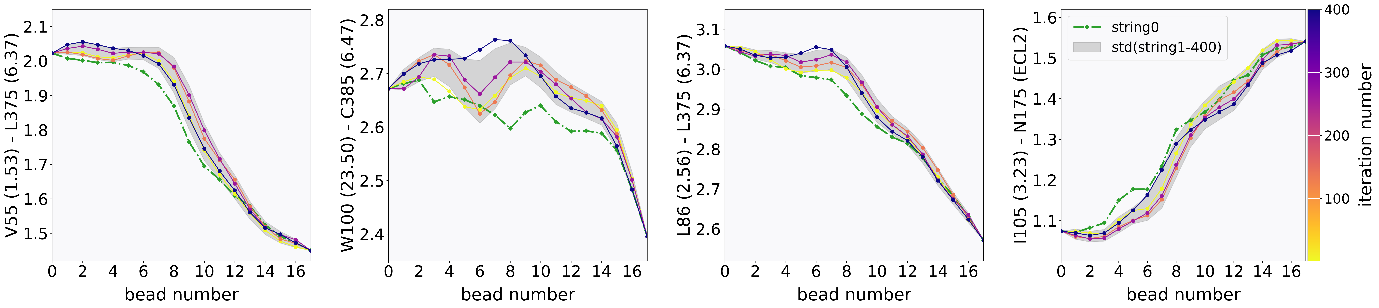


Fig. S13. Strings averaged over hundreds of iterations for unliganded D2R-I66T mutant initiated from the active structure (PDB ID 6VMS). The top 4 important collective variables (CVs) in Table S1 are shown on the y-axis to evaluate the string convergence. The x-axis shows the evolution of the string points towards the inactive state.


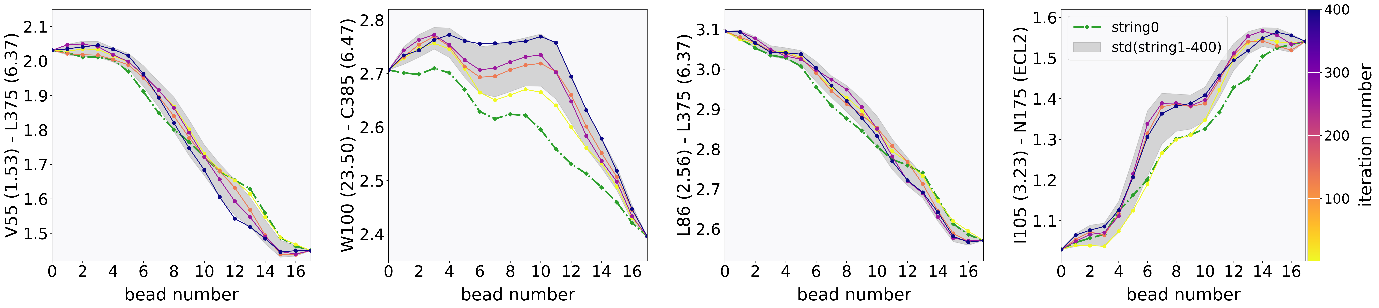


Fig. S14. Strings averaged over hundreds of iterations for unliganded D2R-Y192K mutant initiated from the active structure (PDB ID 6VMS). The top 4 important collective variables (CVs) in Table S1 are shown on the y-axis to evaluate the string convergence. The x-axis shows the evolution of the string points towards the inactive state.


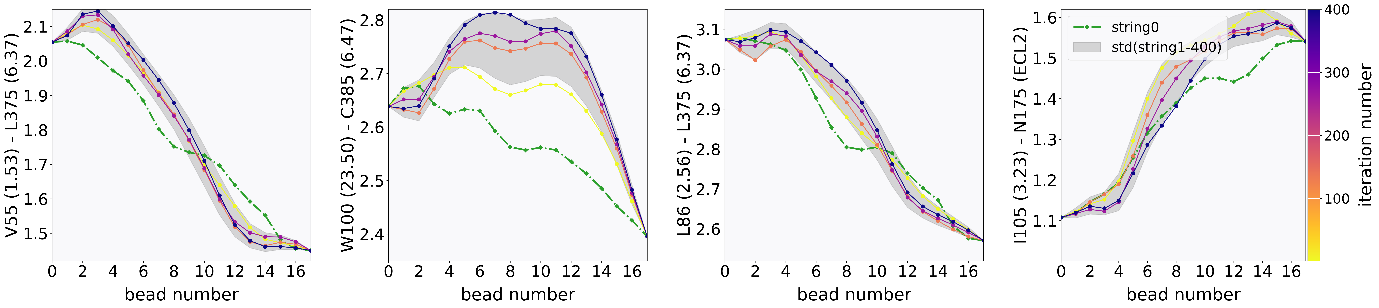


Fig. S15. Strings averaged over hundreds of iterations for unliganded D2R-T372R mutant initiated from the active structure (PDB ID 6VMS). The top 4 important collective variables (CVs) in Table S1 are shown on the y-axis to evaluate the string convergence. The x-axis shows the evolution of the string points towards the inactive state.


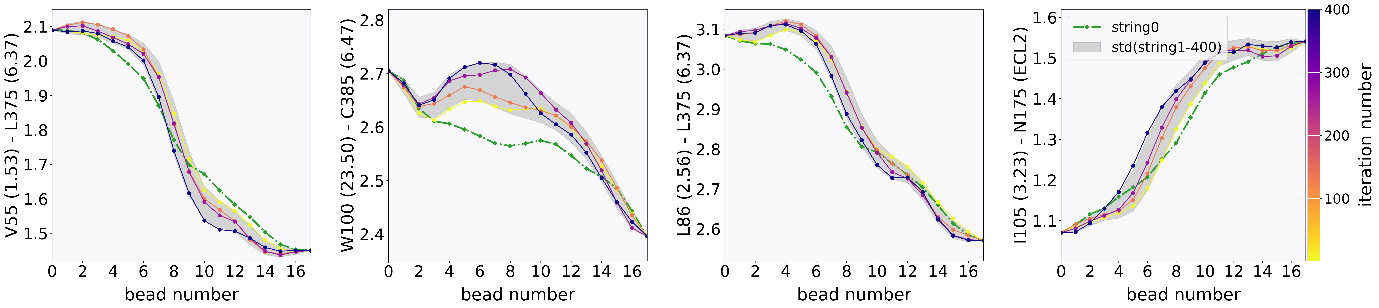


Fig. S16. Strings averaged over hundreds of iterations for unliganded D2R-T372K mutant initiated from the active structure (PDB ID 6VMS). The top 4 important collective variables (CVs) in Table S1 are shown on the y-axis to evaluate the string convergence. The x-axis shows the evolution of the string points towards the inactive state.


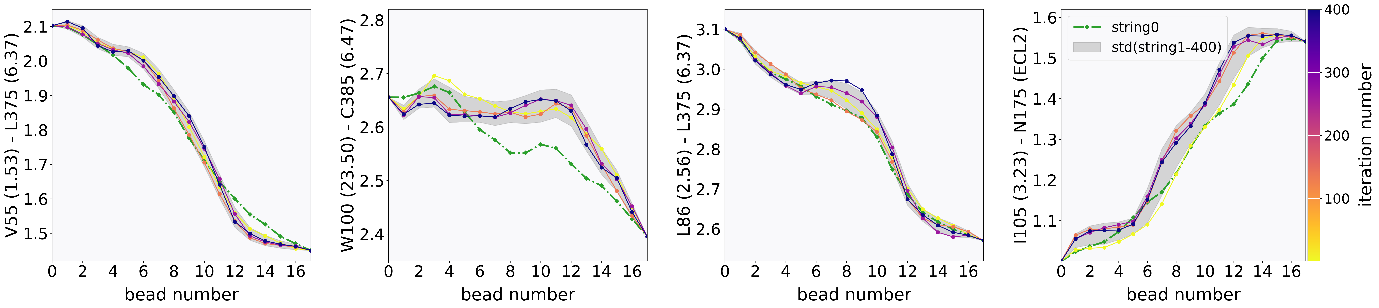


Fig. S17. Strings averaged over hundreds of iterations for unliganded D2R-A376K mutant initiated from the active structure (PDB ID 6VMS). The top 4 important collective variables (CVs) in Table S1 are shown on the y-axis to evaluate the string convergence. The x-axis shows the evolution of the string points towards the inactive state.


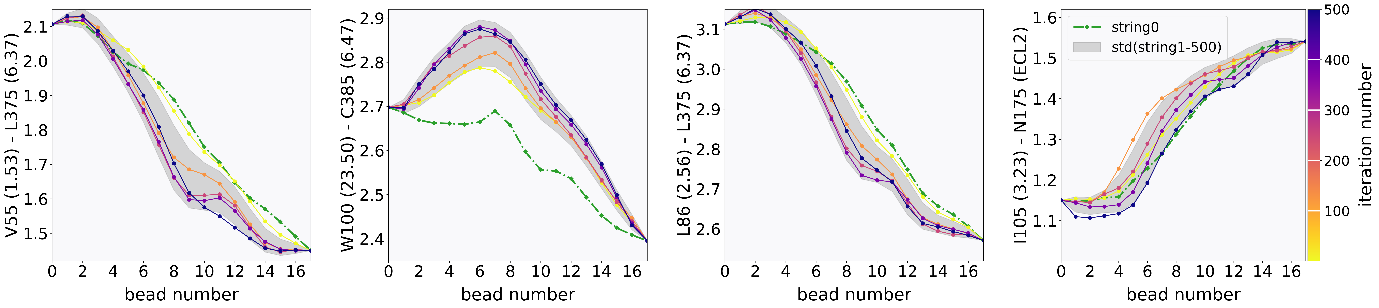


Fig. S18. Strings averaged over hundreds of iterations for unliganded D2R-N418I mutant initiated from the active structure (PDB ID 6VMS). The top 4 important collective variables (CVs) in Table S1 are shown on the y-axis to evaluate the string convergence. The x-axis shows the evolution of the string points towards the inactive state.


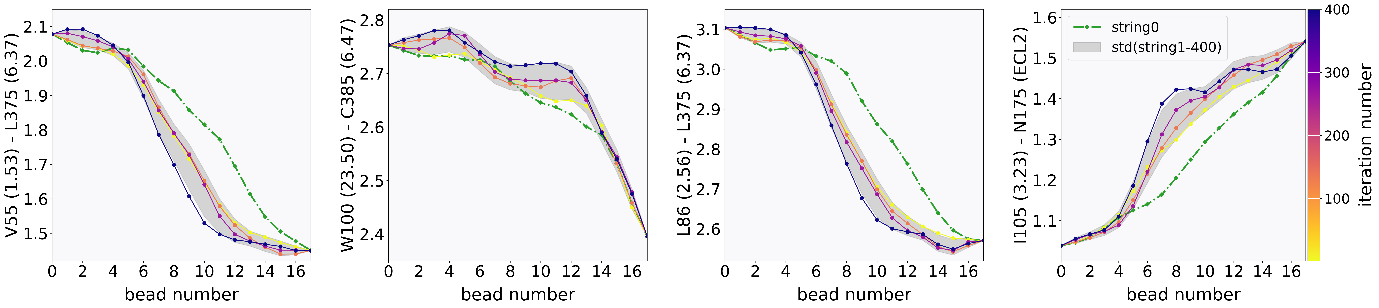


Fig. S19. Strings averaged over hundreds of iterations for unliganded D2R-S419L mutant initiated from the active structure (PDB ID 6VMS). The top 4 important collective variables (CVs) in Table S1 are shown on the y-axis to evaluate the string convergence. The x-axis shows the evolution of the string points towards the inactive state.


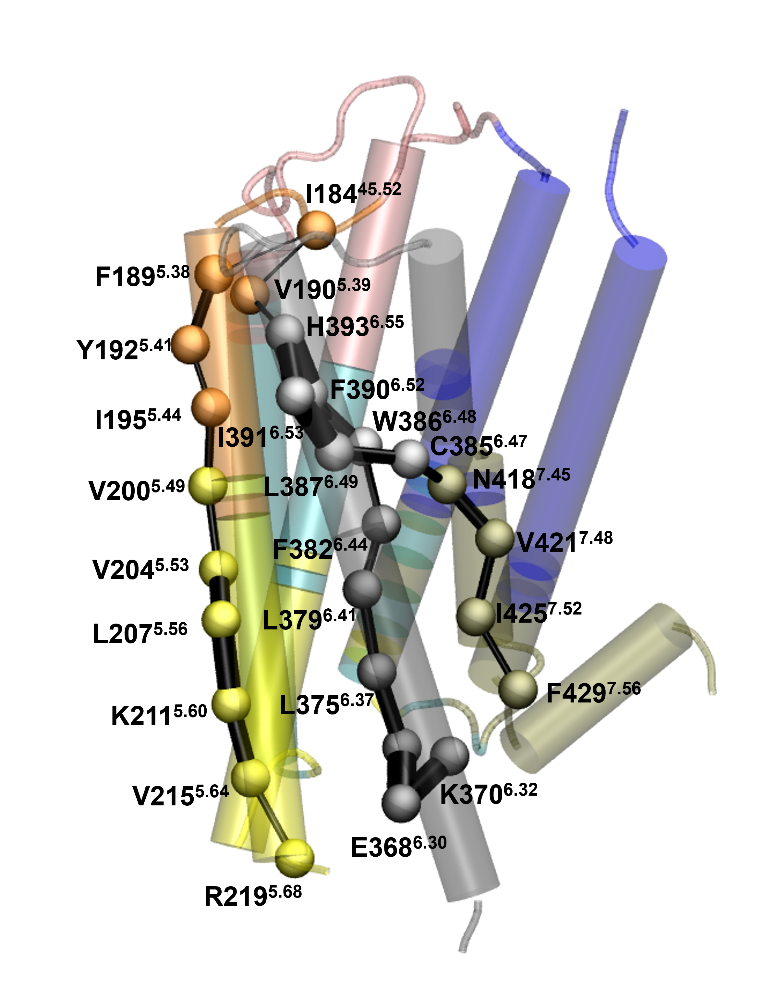


Fig. S20. Mutation-specific allosteric signal pathways from the extracellular to intracellular domains in the mutant T69^2.39^E. Optimal pathways from I184^45.52^ (source node) to R219^5.68^, K370^6.32^ and F429^7.56^ were calculated. Residues involved in pathways are rendered as spheres and colored consistently with communities, and the connecting edges are represented by lines with their width weighted by betweenness.

Table S1. Total simulation time for each D2R system.

| Mutation | PDB structure | Steered MD | String method with swarms of trajectories/iterations | Total simulation time (μs) |
| --- | --- | --- | --- | --- |
| T205^5.54^, M374^6.36^, V378^6.40^, V381^6.43^, and V421^7.48^ | 6VMS (active) | 102 ns | 400 (2.24 μs) | 2.342 |
| I48^1.46^W | 6VMS (active) | 102 ns | 400 (2.24 μs) | 2.342 |
| I48^1.46^Y | 6VMS (active) | 102 ns | 400 (2.24 μs) | 2.342 |
| I48^1.46^A | 6VMS (active) | 102 ns | 400 (2.24 μs) | 2.342 |
| T69^2.39^E | 6VMS (active) | 102 ns | 400 (2.24 μs) | 2.342 |
| I73^2.43^K | 6VMS (active) | 102 ns | 400 (2.24 μs) | 2.342 |
| L125^3.43^K | 6VMS (active) | 102 ns | 400 (2.24 μs) | 2.342 |
| I166^4.56^A | 6VMS (active) | 102 ns | 400 (2.24 μs) | 2.342 |
| I166^4.56^T | 6VMS (active) | 102 ns | 400 (2.24 μs) | 2.342 |
| Y192^5.41^K | 6VMS (active) | 102 ns | 400 (2.24 μs) | 2.342 |
| T372^6.34^K | 6VMS (active) | 102 ns | 400 (2.24 μs) | 2.342 |
| T372^6.34^R | 6VMS (active) | 102 ns | 400 (2.24 μs) | 2.342 |
| A376^6.38^K | 6VMS (active) | 102 ns | 400 (2.24 μs) | 2.342 |
| N418^7.45^I | 6VMS (active) | 102 ns | 500 (2.8 μs) | 2.902 |
| S419^7.46^L | 6VMS (active) | 102 ns | 400 (2.24 μs) | 2.342 |

Table S2. Local functional microswitches used to characterize the free energy landscapes

| Name | Measurement |
| --- | --- |
| RMSD of CWxP | Root-mean-square deviation (RMSD) of C6.47, W6.48, and P6.50 heavy atoms to the active structure 6VMS |
| RMSD of PIF | RMSD of I3.40 and F6.44 heavy atoms to the active structure 6VMS |
| RMSD of NPxxY | RMSD of N7.49, P7.50 and Y7.53 heavy atoms to the active structure 6VMS |
| TM3-TM6 distance | R3.50 – E6.30 Cα distance |

Table S3. Experimental structures of active and inactive D2R and D3R used for structural comparison.

| **Receptor** | | **Ligand** | **Function** | **G Protein** | **Method** | **PDB** | **Resolution** | | **State** |
| --- | --- | --- | --- | --- | --- | --- | --- | --- | --- |
| D2R | rotigotine | | Agonist | Gi/o | cryo-EM | 8IRS | 3.0 | Active | |
| D3R | rotigotine | | Agonist | Gi/o | cryo-EM | 8IRT | 2.7 | Active | |
| D2R | bromocriptine | | Agonist | Gi/o | cryo-EM | 7JVR | 2.8 | Active | |
| D2R | bromocriptine | | Agonist | Gi/o | cryo-EM | 6VMS | 3.8 | Active | |
| D3R | PD128907 | | Agonist | Gi/o | cryo-EM | 7CMV | 2.7 | Active | |
| D3R | pramipexole | | Agonist | Gi/o | cryo-EM | 7CMU | 3.0 | Active | |
| D2R | spiperone | | Antagonist | - | X-ray | 7DFP | 3.1 | Inactive | |
| D2R | haloperidol | | Antagonist | - | X-ray | 6LUQ | 3.1 | Inactive | |
| D2R | rispoeridone | | Inverse agonist | - | X-ray | 6CM4 | 2.9 | Inactive | |
| D3R | eticlopride | | Antagonist | - | X-ray | 3PBL | 2.9 | Inactive | |

Tabel S4. Twelve contact pairs extracted from structural analysis important for the stability of the inactive state with selected residues in bold for *in-silico* residue scanning.

|  | Position 1 | Position 2 |
| --- | --- | --- |
| 1 | **I48^1.46^** | **S419^7.46^** |
| 2 | L65^12.50^ | E432^8.49^ |
| 3 | **T69^2.39^** | R132^3.50^ |
| 4 | **I73^2.43^** | Y426^7.53^ |
| 5 | **L125^3.43^** | F382^6.44^ |
| 6 | R132^3.50^ | E368^6.30^ |
| 7 | R132^3.50^ | **T372^6.34^** |
| 8 | **I166^4.56^** | **Y192^5.41^** |
| 9 | Y209^5.58^ | **A376^6.38^** |
| 10 | F382^6.44^ | **N418^7.45^** |
| 11 | W386^6.48^ | Y416^7.43^ |
| 12 | H389^6.51^ | H393^6.55^ |
